# RNA Interference and BMP-2 Stimulation Allows Equine Chondrocytes Redifferentiation in 3D-Hypoxia Cell Culture Model: Application for Matrix-Induced Autologous Chondrocyte Implantation

**DOI:** 10.3390/ijms18091842

**Published:** 2017-08-24

**Authors:** Rodolphe Rakic, Bastien Bourdon, Magalie Hervieu, Thomas Branly, Florence Legendre, Nathalie Saulnier, Fabrice Audigié, Stéphane Maddens, Magali Demoor, Philippe Galera

**Affiliations:** 1 Normandie Université, UNICAEN, Laboratoire Microenvironnement Cellulaire et Pathologies (MILPAT), équipe Microenvironnement des Pathologies Dégénératives et Fibrotiques (MIPDF), EA 4652/BIOTARGEN EA 7450, UFR Santé, Université de Caen Normandie, 14032 Caen, France; rodolphe.rakic@gmail.com (R.R.); bourdon.bas@laposte.net (B.B.); magalie.hervieu@gmail.com (M.H.); tbranly@gmail.com (T.B.); florence.legendre@unicaen.fr (F.L.) ; magali.demoor@unicaen.fr (M.D.); 2 Vetbiobank, 1 Avenue Bourgelat, 69280 Marcy l’Etoile, France; n.saulnier@vetbiobank.com (N.S.); s.maddens@vetbiobank.com (S.M.); 3 Imaging and Research Centre of Equine Locomotor Disorders (CIRALE, 14430 Goustranville, France), Ecole Nationale Vétérinaire d’Alfort, 94704 Maisons-Alfort, France; fabrice.audigie@vet-alfort.fr

**Keywords:** chondrocyte, tissue engineering, equine model, dedifferentiation, matrix-associated autologous chondrocyte implantation (MACI), BMP-2, hypoxia, siRNA, *Col1a1*, *HtrA1*, type II collagen

## Abstract

As in humans, osteoarthritis (OA) causes considerable economic loss to the equine industry. New hopes for cartilage repair have emerged with the matrix-associated autologous chondrocyte implantation (MACI). Nevertheless, its limitation is due to the dedifferentiation occurring during the chondrocyte amplification phase, leading to the loss of its capacity to produce a hyaline extracellular matrix (ECM). To enhance the MACI therapy efficiency, we have developed a strategy for chondrocyte redifferentiation, and demonstrated its feasibility in the equine model. Thus, to mimic the cartilage microenvironment, the equine dedifferentiated chondrocytes were cultured in type I/III collagen sponges for 7 days under hypoxia in the presence of BMP-2. In addition, chondrocytes were transfected by siRNA targeting *Col1a1* and *Htra1* mRNAs, which are overexpressed during dedifferentiation and OA. To investigate the quality of the neo-synthesized ECM, specific and atypical cartilage markers were evaluated by RT-qPCR and Western blot. Our results show that the combination of 3D hypoxia cell culture, BMP-2 (Bone morphogenetic protein-2), and RNA interference, increases the chondrocytes functional indexes (*Col2a1*/*Col1a1*, *Acan*/*Col1a1*), leading to an effective chondrocyte redifferentiation. These data represent a proof of concept for this process of application, in vitro, in the equine model, and will lead to the improvement of the MACI efficiency for cartilage tissue engineering therapy in preclinical/clinical trials, both in equine and human medicine.

## 1. Introduction

Cartilage defects caused by traumas, focal lesions, or articular overstress, are the leading cause of osteoarthritis (OA) development. This incurable pathology generates pain, walking-related disability, and high morbidity. The poor intrinsic cartilage self-repair is not able to prevent the ineluctable tissue degradation. Chondrocytes, the major cartilage cell type, synthesize an abundant extracellular matrix (ECM), mainly composed of two specific phenotypic markers (type II collagen and aggrecan). During OA, the primary chondrocyte response to the inflammatory microenvironment is a change in its normal secretome, leading to the decreased expression of cartilage specific markers and matrix metalloproteinases synthesis. ECM remodeling occurs, resulting in an ineluctable homeostasis loss. In parallel, chondrocytes dedifferentiate and express the fibrotic type I collagen, leading to fibrocartilage of poor quality.

As in humans, disorders of the equine musculoskeletal system cause considerable economic losses in equine industry [1]. The use of horses for sports makes this model one of the first targets of OA [2]. OA is responsible for up to 60% of lameness, and represents a direct and indirect cost of $15,000/year per horse in the U.S. (compared to $10,000/year per human) [3]. The similarity in epidemiology and physiopathology between humans and horses makes the latter an excellent study model [1,4]. To treat OA, major medical and veterinary prescriptions are mainly composed of analgesic and anti-inflammatory drugs, but these treatments remain palliative. Additionally, advanced OA cannot be treated, while early treatment of cartilage defects can stop its progression.

In the past decades, advances in regenerative medicine have led to the emergence and improvement of new generations of therapies based on tissue engineering [5]. Among these approaches, matrix-induced autologous chondrocyte implantation (MACI) has been established as an attractive and hopeful therapy [6,7] for treating cartilage defects in horses [8,9,10]. Such therapy combining the use of chondrocytes and biomaterials is minimally invasive and shows promising results for cartilage healing, both in humans and horses [11]. Although good outcomes have been reported, MACI still has some limitations and disadvantages. In particular, this technique requires a substantial number of chondrocytes that lose their phenotype during in vitro amplification, a phenomenon called dedifferentiation [12,13,14]. In this sense, during expansion in monolayers, the chondrocytes dedifferentiate into fibroblast-like cells, and stop expressing cartilage specific markers, such as type II collagen or aggrecan [15,16]. Concomitantly, they secrete catabolic mediators and regulators [17,18,19]. The imbalance between anabolism and catabolism leads to cell homeostasis disruption [20]. Furthermore, following long term culture, dedifferentiated chondrocytes produce fibrotic type I collagen in an exacerbated manner [13]. Consequently, the newly synthesized cartilage substitute does not own the same biomechanical and physiological properties compared to the hyaline articular cartilage [21].

Two major in vitro strategies have been investigated in order to optimize the mature chondrocyte phenotype for MACI: the maintenance of the mature chondrocyte phenotype during extensive cell culture [22], or the chondrocyte redifferentiation after extensive cell culture. The present study particularly focused on the improvement of the redifferentiation process by finely controlling some cellular microenvironment parameters, which are key regulators of chondrocytes’ behaviour [22].

It was demonstrated that high cell density three-dimensional (3D) cultures, in combination with hypoxia, support redifferentiation of dedifferentiated articular chondrocytes. Hypoxia is known to act on type II and type X collagen expression [23]. Indeed, low oxygen tension during chondrocyte culture is able to stabilize cell phenotype by favoring the mature chondrocyte phenotype and preventing hypertrophy [24,25]. Additionally, cell culture with selective growth factors and cytokines appropriately supplemented, plays a major role in the regulation of anabolic and catabolic chondrocyte activities. In particular, members of the transforming growth factor-β (TGF-β) superfamily may exert a dual role on chondrocyte development. Among them, bone-morphogenetic-proteins (BMPs) such as BMP-2 is used as a pro-chondrogenic factor at low concentration [26], allowing the recovery of type II collagen and aggrecan syntheses by chondrocytes [27,28]. Although BMP-2 contributes to the redifferentiation of articular chondrocytes, it also simultaneously induces type I collagen chondrocyte expression [29]. This overexpression of both collagen isoforms prevents high biological quality of extracellular matrix (ECM) synthesis.

On the other hand, BMP-2 cellular responses can be affected by high-temperature requirement A serine peptidase 1 (HtrA1), a serine protease. Indeed, Htra1 is able to inhibit TGF-β family signalling via proteolytic activity, and can play a role in BMP-mediated redifferentiation of chondrocytes [20,30,31]. Furthermore, among the catabolic events, the serine-protease Htra1 seems to have important roles in OA development, ECM remodelling and cell fate [30,31,32,33,34]. This is corroborated by the fact that HtrA1 is expressed by chondrocytes, is up-regulated during OA, and is present at high levels in synovial fluid, and seems, as a consequence, to contribute to OA development and change in cartilage composition [35,36].

**Figure 1:**
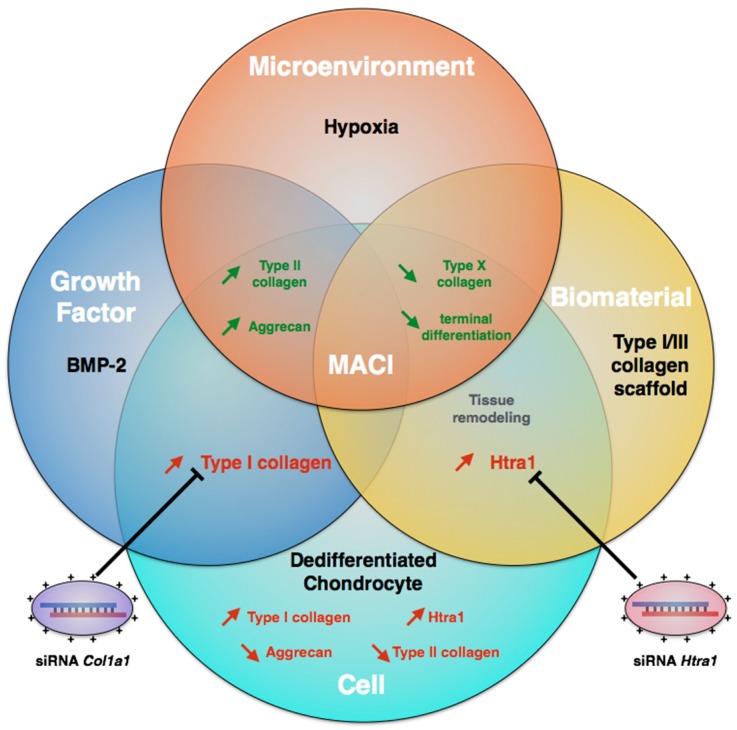
Improvement of BMP-2 (Bone morphogenetic protein-2) mediated matrix-associated autologous chondrocyte implantation (MACI) process based on RNA interference. MACI is a tissue engineering method involving different parameters: biomaterial, cells, and exogenous stimuli (growth factors and cellular microenvironment). To induce chondrocyte redifferentiation, chondrocytes are cultured in 3D-Hypoxia conditions and treated with BMP-2. To counteract side effects of BMP-2 mediated redifferentiation, RNA interference targeting *Col1a1* and *Htra1* can be used to avoid fibrotic and catabolic evolution of the MACI process. The red arrows represent the atypical comportment of chondrocyte and deleterious effects whereas green arrows represent positive effects on the specific expression of hyaline articular cartilage components.

Autograft synthesis, based on chondrocyte redifferentiation mediated by BMP-2, leads to fibrotic and pro-catabolic cartilage substitutes. To limit this problem, specific inhibition of atypical markers of fibrocartilage is necessary. Some studies have shown that RNA interference can finely modulate the chondrocyte phenotype in vitro, but these investigations are all based on the fundamental aspect of cell biology, and not on the context of applied biology [37,38,39]. Here, we used a multifactorial strategy, based on the use of 3D cell culture in type I/III collagen sponges and BMP-2 stimulation under hypoxia condition [40]. To counteract the up-regulation of type I collagen induced by BMP-2, and the Htra1 induction in culture, we used a specific and transient RNA interference approach, targeting *Col1a1* and *Htra1* mRNA (Figure 1).

We have extensively analyzed the expression of cartilage specific markers, catabolic enzymes, and in particular, focused on the fibrotic aspect of chondrocytes during redifferentiation. Finally, the quality of the ECM cartilage synthesis was especially determined by the consideration of the functional indexes *Col2a1*/*Col1a1* and *Acan*/*Col1a1*. Our results demonstrated that this strategy allows type II collagen synthesis, while the level of type I collagen expression was drastically reduced in the horse model. This suggests that the use of siRNA could be a valuable approach for the tissue engineering of functional articular cartilage.

## 2. Results

### 2.1. Loss of Equine Articular Chondrocyte Phenotype during Amplification

**Figure 2:**
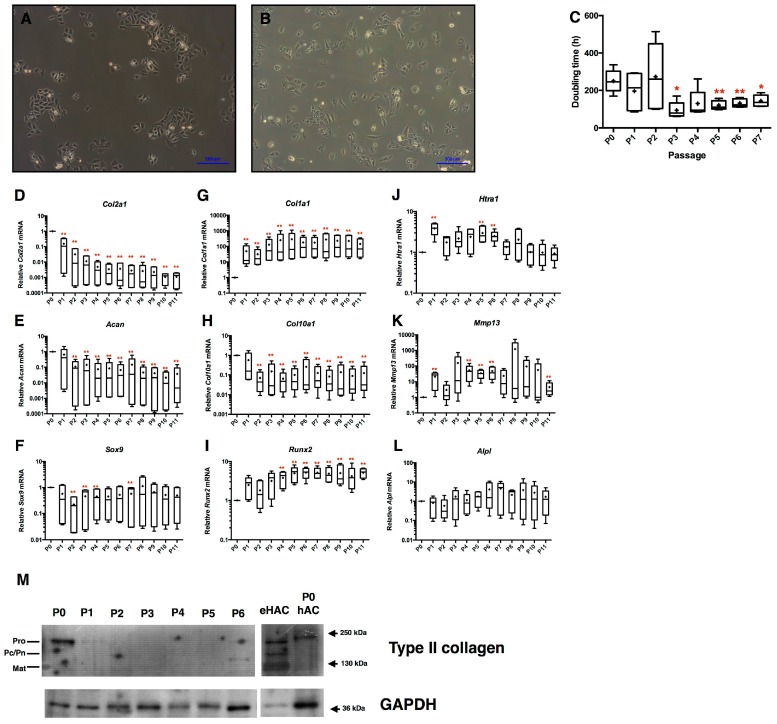
Equine articular chondrocytes become more proliferative and lose their specific expression profile during dedifferentiation. Phase-contrast microscopy of primary chondrocytes (**A**) and P3 chondrocytes (**B**) cultured in monolayers (magnification ×10). eAC isolated from equine cartilage were dedifferentiated during 11 passages in monolayer (*n* = 3). At each passage, doubling times of eAC are determined (**C**) and relative mRNA expression of *Col2a1*, *Col1a1*, *Htra1*, *Acan*, *Col10a1*, *Mmp13*, *Sox9*, *Runx2* and *Alpl* (respectively **D**–**L**) were analyzed by RT-qPCR. Statistically significant differences are determined using the Mann–Whitney test (* *p* < 0.05, ** *p* < 0.01). (**M**) Protein extracts were analyzed in Western blots for type II collagen versus GAPDH. Representative blots are shown. Equine hyaline articular chondrocytes (eHAC) and primary (P0) human articular chondrocytes (hAC) protein extracts show different levels of type II maturation forms such as type II procollagen (pro), with only C- or N-terminal propeptides (Pc/Pn) and the mature doubly cleaved form (mat). The weak signal of eHAC GAPDH (glyceraldehyde-3-phosphate dehydrogenase) is caused by the low proportion of cells in native hyaline cartilage facing the global proteins content of this tissue.

To evaluate chondrocyte phenotype recovery after amplification, eAC isolated from equine cartilage were cultured for 11 passages. During the subculture, eAC lose their classical polygonal shape after only 2 passages, and exhibit cytoplasmic elongations characteristic of fibroblastic-like cells (Figure 2A,B). Concomitantly, the doubling time of eAC decreases drastically by 3-fold at P3 compared to P0, and then remains constant until P7 (Figure 2C). During the sub-cultures, the cartilage specific markers were lost. Indeed, the relative mRNA levels of *Col2a1*, *Acan*, and *Sox9* decrease overall from P2 (Figure 2D–F; respectively 33-fold, 10-fold, and 5-fold between P0 and P2) as well as *Col10a1* (17-fold between P0 and P2) (Figure 2H). *Col2a1* mRNA level was strongly repressed after P2. In parallel, the steady-state amounts of the atypical *Col1a1* mRNA strongly increase (43-fold at P1 versus P0) (Figure 2G). Data were normalized to P0 cells in these experiments, and very weak amounts of *Col1a1* mRNA were nonetheless detected in primary cells (P0) in some cases. We supposed that enzymatic digestion for cell isolation induces a slight stress to chondrocytes, which express *Col1a1* mRNA de novo. However, no detection of type I collagen protein was observed in native cartilage protein extracts used as control (data not shown). The catabolic markers *Htra1* and *Mmp13* are transiently more highly expressed, from P4 to P6 (Figure 2J,K). Meanwhile, *Runx2* mRNA is strongly overexpressed after P4 (Figure 2I), whereas *AlpI* mRNA is not modulated (Figure 2L). Only primary eAC (P0) is able to express type II procollagen (Figure 2M).

### 2.2. Combination of 3D-Cell Culture, Hypoxia, and BMP-2 Treatment Allows the Recovery of Cartilage Specific Marker Expression by eAC, but Does Not Abolish the Fibrotic and Catabolic Components

**Figure 3:**
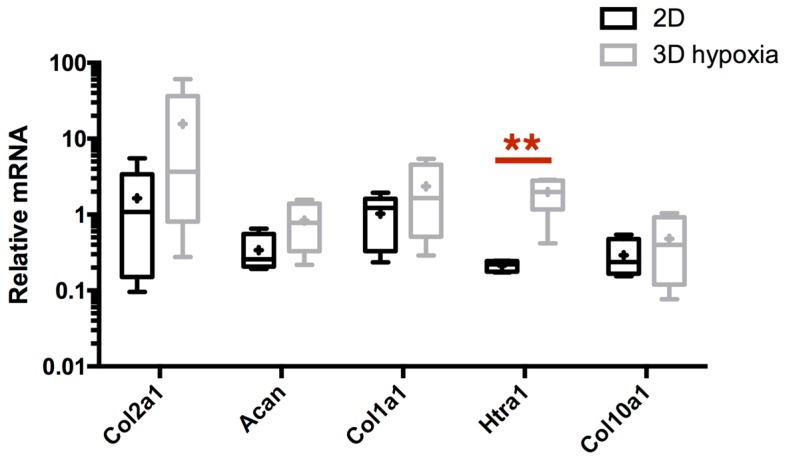
eAC 3D culture in type I/III collagen sponges under hypoxia induces some changes in the expression of cartilage markers of interest. After eAC dedifferentiation during 2 passages, cells were trypsinized and seeded in type I/III collagen sponges. Cells were placed in hypoxia during 7 days (3D Hypoxia). Cells were subjected to relative mRNA expression analysis by RT-qPCR and compared to P3 eAC cultured in monolayers in normoxia (2D). All the data are normalized versus eAC seeded in sponges and arrested after 16 h of incubation, and presented as the relative expression of each gene. Box plots represent five independent experiments performed in triplicate. Statistically significant differences are determined using the Mann–Whitney test (** *p* < 0.01).

Because the microenvironment strongly influences chondrocyte phenotype, our strategy intended to be closer to the cellular context observed in native hyaline articular cartilage. In the latter, oxygen tension oscillates between 6% and 1% O_2_ [41]. Thus, after chondrocyte dedifferentiation, cells were cultured for 7 days in type I/III collagen sponges under hypoxia (2–4% O_2_). First, we observed that only *Htra1* mRNA expression is significantly enhanced by 3D/hypoxia cell culture, compared to the 2D culture in normoxia (10-fold increase between 2D and 3D/hypoxia conditions) (Figure 3). Differences between monolayer cultured cells and 3D basal culture conditions show that the latter, which induces rapid *Htra1* mRNA increase, is reminiscent of its homology with stress-induced HtrA proteases family [42]. Moreover, 3D culture weakly enhances the transcription of *Col2a1*, *Acan*, and *Col1a1*, even though it is not statistically significant, without modulating the steady-state amount of *Col10a1* mRNA.

**Figure 4:**
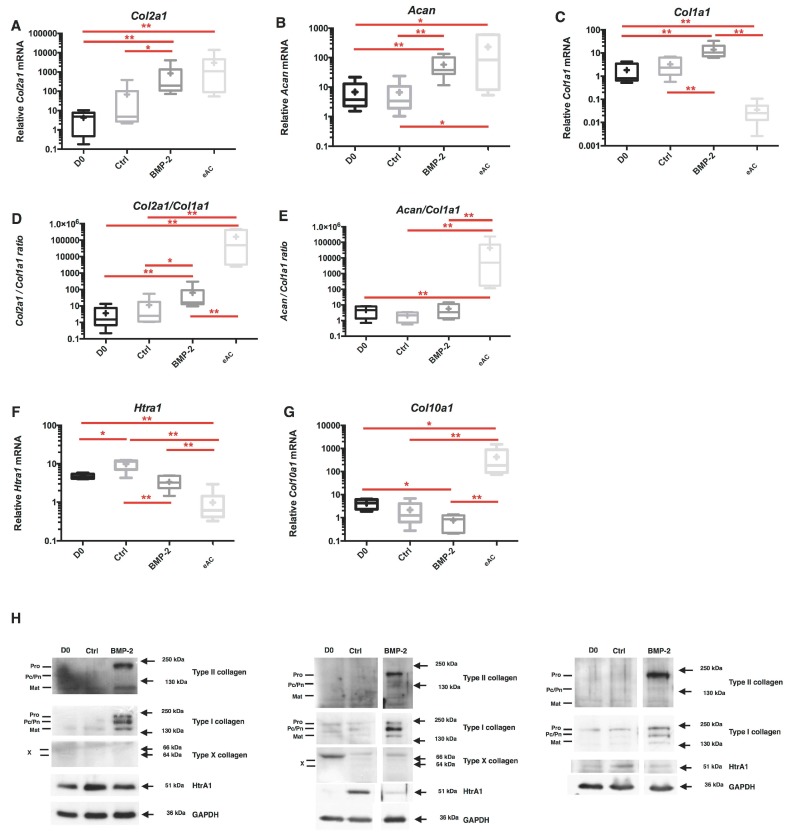
3D culture, hypoxia and BMP-2 stimulation of eAC allow the expression recovery of cartilage specific markers. After eAC dedifferentiation during 2 passages, cells were trypsinized and seeded in type I/III collagen sponges. Cells were incubated in hypoxia and treated with BMP-2 (BMP-2) (50 ng/mL) or not (Ctrl) during 7 days. Relative mRNA amounts encoding *Col2a1* (**A**), *Acan* (**B**), *Col1a1* (**C**), *Htra1* (**F**), *Col10a1* (**G**) were determined by RT-qPCR. The quality indexes of the neocartilage *Col2a1/Col1a1* (**D**) and *Acan/Col1a1* (**E**) are calculated. mRNA extracts obtained from equine articular chondrocytes (eAC) released from cartilage after overnight enzymatic digestion are used as controls. D0: Cells seeded in sponges and arrested after 16 h of incubation. All results are normalized versus eAC cultured in monolayer, and presented as the relative expression of each gene. Box plots represent six independent experiments performed in triplicate. Statistically significant differences are determined using the Mann–Whitney test (* *p* < 0.05, ** *p* < 0.01); (**H**) protein extracts were analyzed in Western blots for type II, type I, and type X collagens, and HtrA1 versus GAPDH. Representative blots are shown (*n* = 5). Different levels of type II and type I collagen maturation forms are indicated, such as type II procollagen (pro), with only C- or N-terminal propeptides (Pc/Pn) and the mature doubly cleaved form (mat). The 64 kDa type X collagen band represents the signal peptide-cleaved form.

To try to allow for better recovery in the expression of chondrocyte-specific markers, eAC were incubated in the presence of BMP-2 (50 ng/mL) and 3D/hypoxia cultured cells were used as control (Ctrl). In these experimental conditions, BMP-2 strongly enhances the *Col2a1* and *Acan* mRNA expression (respectively 219-fold and 8-fold compared to D0), but also *Col1a1* mRNA (8-fold) (Figure 4A–C). The effect on *Col1a1* mRNA explains the low change of *Col2a1*/*Col1a1* ratio (5-fold increase) and *Acan/Col1a1 ratio* (not significant) used as functional indexes of the cartilage ECM (Figure 4D,E). Interestingly, while relative *Htra1* mRNA increases during the 7-day period of culture (*p* < 0.05 for Ctrl compared to D0), the BMP-2 treatment is able to significantly decrease the steady-state amounts of *Htra1*, by 3-fold compared to Ctrl (Figure 4F). Likewise, the relative mRNA levels of *Col10a1* are repressed by BMP-2 treatment. Furthermore, no significant modulation of total protein content was observed upon BMP-2 incubation. Nevertheless, an increasing trend seems to be detectable (Figure S1).

eACs directly isolated from articular cartilage can be considered to be the most efficient control in terms of phenotype quality. Our results show that even if the use of BMP-2 leads to comparable mRNA steady-state levels of *Col2a1* and *Acan* between the in vitro cultured chondrocytes and fresh cells, the functional indexes remain incomparable due to the absolute lack of basal *Col1a1* expression in eACs from native cartilage. The poor ECM quality induced by BMP-2 to generate a hyaline cartilaginous substitute is represented by the persistence of type I collagen and HtrA1 expression at the protein level, whereas type II collagen synthesis is recovered and type X collagen seemed to be undetectable (Figure 4H).

### 2.3. Design of siRNA

**Figure 5:**
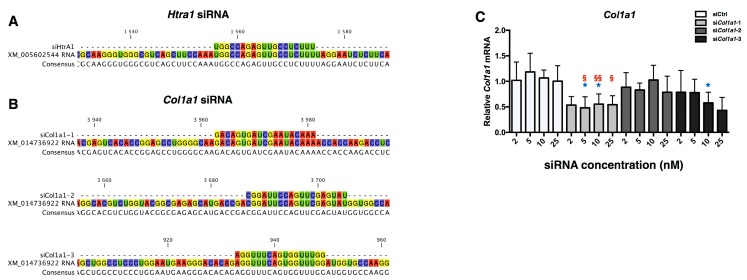
siRNA design and efficiency determination of *Col1a1* siRNA. Sequence alignment between *Htra1* siRNA (**A**) or the three *Col1a1* siRNA (**B**) and their respective mRNA targets are presented. XM_005602544: predicted *Equus caballus* Htra serine peptidase 1 (*Htra1*), mRNA NCBI reference sequence. XM_014736922: predicted *Equus caballus* collagen, type I α1 (COL1A1), mRNA NCBI reference sequence. Software: CLC sequence viewer. After eAC dedifferentiation during 2 passages, cells were trypsinized and seeded in type I/III collagen sponges. Cells were transfected by different concentrations of three *Col1a1* siRNA in hypoxia and treated with BMP-2 (50 ng/mL) for 7 days (**C**). The relative expression of *Col1a1* mRNA was determined by RT-qPCR. Cells transfected with a negative control siRNA were used as control (siCtrl). All the results are normalized against BMP-2 treated cells without transfection, and are presented as the relative expression of each gene. The data represent three independent experiments performed in triplicate. Statistically significant differences between siCtrl and siCol1a1 transfected cells at the same siRNA concentration are determined using unpaired (* *p* < 0.05) or paired T-test (^§^ *p* < 0.05, ^§§^ *p* < 0.01).

Due to the fibrotic and catabolic effects of BMP-2 induced MACI process in eAC, a strategy of RNA interference has been developed on the basis of the study performed with human articular chondrocytes [40]. However, to achieve the proof of concept in horse chondrocytes, there is need for a technological transfer towards the equine model. In fact, new designs of siRNA and dose-effects of each siRNA have been performed. Equine *Htra1* siRNA (si*Htra1*) has been previously designed and functionally tested, and the data demonstrate that it provokes an efficient inhibition of its target (Figure 5A and data not shown). More attention was devoted to *Col1a1* siRNA (si*Col1a1*) efficiency evaluation, due to the complex transcriptional and post-transcriptional regulation of this gene.

First, three si*Col1a1* were designed (Figure 5B), and different siRNA concentrations were evaluated (2, 5, 10, 25 nM) on eAC during redifferentiation mediated by BMP-2. A negative control siRNA (siCtrl) was used at the same concentrations as control, to evaluate off-target effects. As shown in Figure 5C, only si*Col1a1*-2 is able to inhibit *Col1a1* mRNA amounts, by more than 50% for all the concentrations, compared to siCtrl at the same concentration. No effects of siCtrl or si*Col1a1* at the same concentrations have been observed on the steady-state amounts of *Col2a1* and *Htra1* (Figure S2). In the following experiments, to take into consideration of the inter-cellular strains variabilities, a concentration of 5 nM for each siRNA was used to obtain an effective mRNA interference.

### 2.4. RNA Interference Targeting Col1a1 Improves eAC Specific Phenotypic Profile during the MACI Process

**Figure 6:**
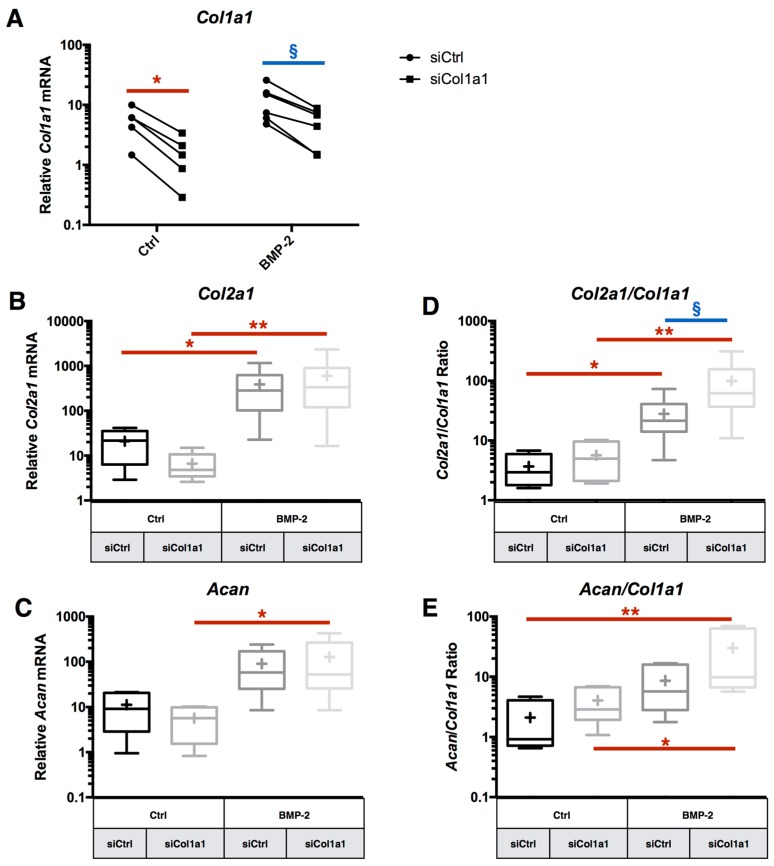
RNA interference targeting *Col1a1* is able to enhance transcriptome quality of chondrocytes. After eAC dedifferentiation during 2 passages, cells were trypsinized and seeded in type I/III collagen sponges. Cells were transfected by 5 nM of *Col1a1* siRNA (si*Col1a1*) in hypoxia and treated with BMP-2 (50 ng/mL) (BMP-2) or not (Ctrl), for 7 days. Relative mRNA levels of *Col1a1* (**A**), *Col2a1* (**B**), and *Acan* (**C**) were determined by RT-qPCR. The quality indexes of the neocartilage *Col2a1*/*Col1a1* (**D**) and *Acan*/*Col1a1* (**E**) are calculated. siCtrl represents cells transfected with a negative control siRNA. All the data are normalized versus eAC cultured in monolayer, and are presented as the relative expression of each gene. Box plots represent five independent experiments performed in triplicate. Statistically significance differences are determined using the Mann–Whitney test (* *p* < 0.05, ** *p* < 0.01) or the Wilcoxon signed-rank test (^§^ *p* < 0.05).

si*Col1a1* transfection inhibits *Col1a1* mRNA expression with or without BMP-2 treatment, respectively 72% and 57% of inhibition, compared to the respective siCtrl conditions (Figure 6A). The experiments, taken one by one, have demonstrated that if more *Col1a1* mRNA is expressed in eAC, then si*Col1a1* is more effective (Figure S3). By contrast, si*Col1a1* induced no significant effect on the relative expression of *Col2a1*, *Acan*, *Mmp13*, and *Htra1* (Figure 6B,C and Figure S4). The biological gain of *Col1a1* mRNA inhibition is visible on the functional indexes *Col2a1*/*Col1a1* and *Acan*/*Col1a1* (Figure 6D,E). Thus, the *Col2a1*/*Col1a1* ratio is increased by 27-fold under si*Col1a1* + BMP-2 treatment, compared to siCtrl without BMP-2 treatment, vs. 8-fold for the siCtrl eAC + BMP-2 condition. The same trend is also observed for the *Acan*/*Col1a1* ratio.

### 2.5. RNA Interference Targeting Htra1 Can Modulate the eAC Catabolic Process during Chondrocyte Redifferentiation

**Figure 7:**
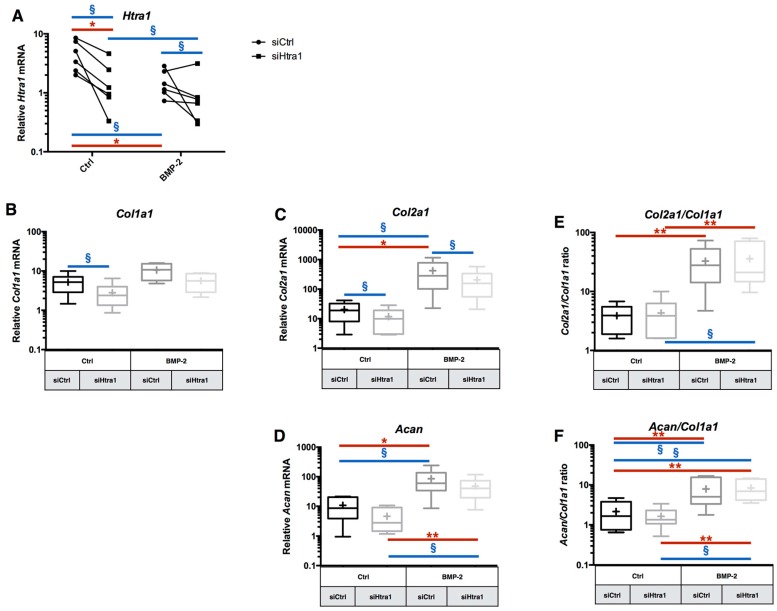
Effects of RNA interference targeting *Htra1* on the expression of this atypical marker during chondrocyte redifferentiation. After eAC dedifferentiation during 2 passages, cells were trypsinized and seeded in type I/III collagen sponges. Cells were transfected by 5 nM of *Htra1* siRNA (si*Htra1*) in hypoxia and treated with BMP-2 (50 ng/mL) (BMP-2) or not (Ctrl) for 7 days. Relative mRNA expression of *Htra1* (**A**), *Col1a1* (**B**), *Col2a1* (**C**), and *Acan* (**E**) were determined by RT-qPCR. The quality indexes of the neocartilage *Col2a1*/*Col1a1* (**D**) and *Acan*/*Col1a1* (**F**) were also calculated. siCtrl represents cells transfected with a negative control siRNA. All the results are normalized versus eAC cultured in monolayer, and presented as the relative expression of each gene. Box plots represent five independent experiments performed in triplicate. Statistically significant differences are determined using the Mann–Whitney test (* *p* < 0.05, ** *p* < 0.01) or the Wilcoxon signed-rank test (^§^ *p* < 0.05).

*Htra1* siRNA treatment provokes a decrease on its mRNA in eAC compared to siCtrl treatment. Indeed, a 64% inhibition by the *Htra1* siRNA is observed when the cells are not treated with BMP-2, and 34% for BMP-2 treated cells (Figure 7A). Even if the siRNA inhibition of *Htra1* is present for the independent experiments (except for one), it seems that this inhibition is lower when the cells are treated with BMP-2. This can be explained by the fact that BMP-2 already decreases *Htra1* mRNA at a comparable basal level of transcription observed in cells cultured as monolayers. Thus, a higher inhibition can be technically difficult to obtain in eAC. Additionally, no significant effect is detected on the relative mRNA expression of *Acan* and *Mmp13* mRNA after si*Htra1* transfection (Figure 7D and Figure S5). Interestingly, si*Htra1* induces a significant decrease of the relative amounts of *Col1a1* mRNA in the absence of BMP-2, and it provoked an inhibition of *Col2a1* mRNA, with or without BMP-2 (Figure 7B,C). Nevertheless, the functional indexes *Col2a1*/*Col1a1* and *Acan*/*Col1a1* are not significantly affected by the si*Htra1* treatment (Figure 7E,F).

### 2.6. RNA Interference during the MACI Process Increases the Quality of the ECM Synthesized by eAC 

**Figure 8:**
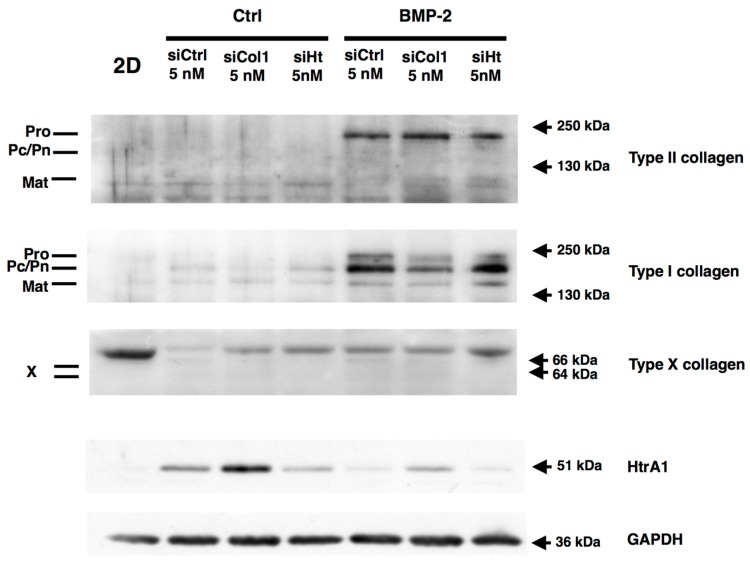
RNA interference targeting *Col1a1* and *Htra1* is able to decrease the expression of these atypical markers during chondrocyte redifferentiation. After eAC dedifferentiation during 2 passages, cells were trypsinized and seeded in type I/III collagen sponges. Cells were transfected by 5 nM of *Col1a1* siRNA (si*Col1*) or *Htra1* siRNA (*siHt*) in hypoxia, and treated, or not, with BMP-2 (50 ng/mL) for 7 days. siCtrl represents cells transfected with negative control siRNA. 2D: P3 eAC cultured in monolayer in normoxia. Protein extracts were analyzed by Western blots for type II, type I, and type X collagens, and HtrA1 versus GAPDH. Representative blots are shown (*n* = 5). Different levels of type II and type I collagen maturation forms are indicated, such as type II procollagen (pro), with only C- or N- terminal propeptides (Pc/Pn), and the mature doubly cleaved form (mat). The 64 kDa type X collagen band represents signal peptide-cleaved form.

At the protein level, the *Col1a1* siRNA caused a decrease of total type I collagen signal, and procollagen expression is strongly repressed (Figure 8). This is consistent with the chronological maturation of type I collagen, and it can be supposed that procollagen is the first isoform affected during inhibition of the *Col1a1* mRNA. Htra1 synthesis is strongly inhibited by BMP-2, and such an effect seems to be sufficient to decrease its expression during eAC cartilaginous substitute generation. Nevertheless, *Htra1* interference totally abolishes Htra1 synthesis when the BMP-2 effect alone was not sufficient, and only in the BMP-2 treated cells (Figure S6). It appears that si*Htra1* transfection affects the BMP-2 redifferentiation effect, since a slight decrease of type I collagen and type II collagen compared to the siCtrl condition is observed. Type X collagen was observed as trace amounts in siCtrl samples.

Overall, these data demonstrate that *Col1a1* interference, combined with BMP-2 and hypoxia in eAC culture, can enhance the quality of the ECM produced by chondrocytes. Even though the si*Htra1* can further abolish its targeted mRNA, the inhibition during BMP-2 redifferentiation seems to be almost totally effective for enhancement of the mature chondrocyte specific phenotype.

### 2.7. Chondrocyte Redifferentiation Mediated by BMP-2 Can Be Modulated by Both Col1a1 and Htra1 siRNAs

To clarify the impact of the different culture parameters, combination of both *Col1a1* and *Htra1* siRNAs was used during the neocartilage substitute generation (3D-hypoxia cell culture and BMP-2 treatment).

**Figure 9:**
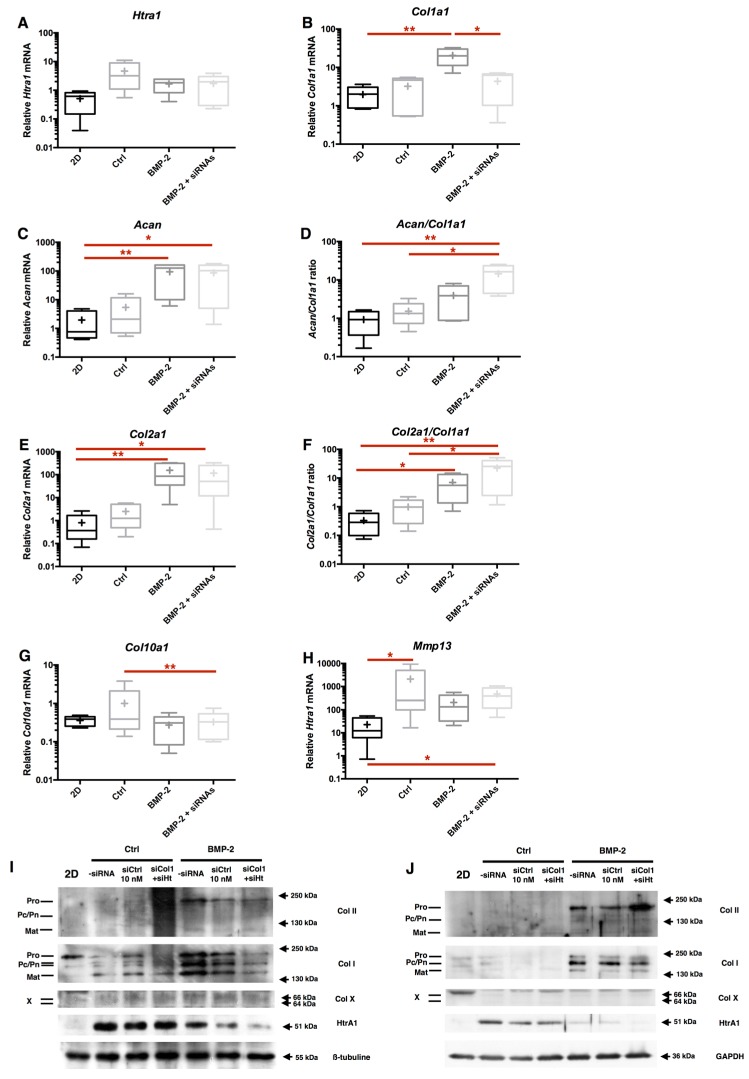
RNA interference targeting *Col1a1* and *Htra1* induces changes in BMP-2 eAC response. After eAC dedifferentiation during 2 passages, cells were trypsinized and seeded in type I/III collagen sponges. Cells were transfected by 5 nM of both *Col1a1* siRNA and *Htra1* siRNA (siRNAs) in hypoxia and treated with BMP-2 (50 ng/mL) for 7 days. Ctrl represents cells which were not transfected and not treated by BMP-2. 2D represents P3 eAC cultured in monolayer in normoxia. Relative mRNA amounts of *Htra1* (**A**), *Col1a1* (**B**), *Acan* (**C**), *Col2a1* (**E**), *Col10a1* (**G**), and *Mmp13* (**H**) were determined by RT-qPCR. The quality indexes of the neocartilage *Acan*/*Col1a1* (**D**) and *Col2a1*/*Col1a1* (**F**) are calculated. Box plots represent five independent experiments performed in triplicate. Statistically significant differences are determined using the Mann–Whitney test (* *p* < 0.05, ** *p* < 0.01) or the Wilcoxon signed-rank test. Protein extracts were analyzed in Western blots for type II, type I, and type X collagens, and HtrA1 versus GAPDH. Two representative blots are shown (*n* = 5) (**I**,**J**). Different levels of type II and type I collagen maturation forms are indicated, such as type II procollagen (pro), with only C- or N-terminal propeptides (Pc/Pn) and the mature doubly cleaved form (mat). The 64 kDa type X collagen band represents signal peptide-cleaved form.

The double siRNA interference was efficient on their respective targets by strongly inhibiting atypical *HtrA1* and *Col1a1* expressions mediated by BMP-2 (Figure 9A,B). Both siRNA treatments show no differences in the relative amounts of *Acan* and *Col2a1* compared to BMP-2 treated cells. The global quality of ECM markers is enhanced, as demonstrated, by the evaluation of *Acan*/*Col1a1* and *Col2a1*/*Col1a1* ratios, when we compare the double interference strategy + BMP-2 with BMP-2 treatment only (Figure 9D,F). At the protein level, the siRNAs effects are also effective on the global type I collagen and HtrA1 syntheses, thereby improving the quality of the neo-synthesized chondrocyte ECM (Figure 9I,J).

**Figure 10:**
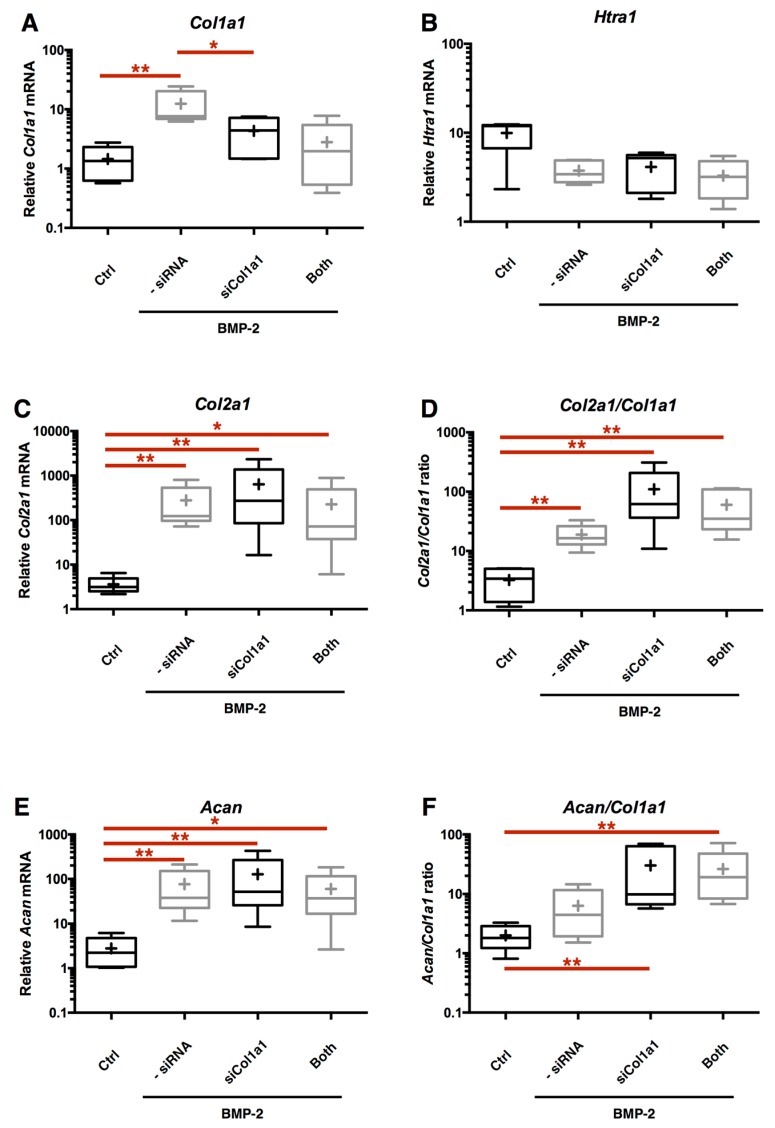
No benefit is detected in the *Col2a1*/*Col1a1* and *Acan*/*Col1a1* ratios during co-transfection of both *Col1a1*-*Htra1* siRNAs, compared to the si*Col1a1* alone. After eAC dedifferentiation during 2 passages, cells were trypsinized and seeded in type I/III collagen sponges. Cells were placed in hypoxia and treated with BMP-2 (50 ng/mL) for 7 days. eAC were transfected with si*Col1a1*, a combination of si*Col1a1* and si*Htra1* (Both), or not transfected (-siRNA). Relative mRNA amounts of *Col1a1* (**A**), *Htra1* (**B**), *Col2a1* (**C**), and *Acan* (**E**) were determined by RT-qPCR. All the data are normalized versus eAC cultured in monolayer, and presented as the relative expression of each gene. The quality indexes of the neocartilage *Col2a1*/*Col1a1* (**D**) and *Acan*/*Col1a1* (**F**) are presented. Box plots represent five independent experiments performed in triplicate. Statistically significant differences are determined using the Mann–Whitney test (* *p* < 0.05, ** *p* < 0.01).

Nevertheless, no further gain in the quality of the neocartilage substitute ECM has been detected between both *HtrA1* and *Col1a1* siRNA transfection versus si*Col1a1* transfection only (Figure 10A–F). Thus, even if this double RNA interference shows its feasibility in knocking down these atypical markers, this strategy does not seem to be better than the *Col1a1* mRNA interference only, which also has the advantage of being more accurate and less stressful for the cells by limiting exogenous genetic material incorporation.

## 3. Discussion

Chondrocytes remain the best cell source used in cartilage tissue engineering. Although new advanced therapies based on MACI are actively developed, like the use of MSCs (Mesenchymal stromal cells), some basic knowledge must be developed for tissue engineering use [43]. Previous studies achieved in the laboratory show that MSCs can be a promising source, as they present a high chondrogenic potential; however, a high level of type I collagen synthesis is also detected [44]. For this reason, in the case of chondrocytes and MSCs, particular attention must be given to the fibrotic aspect for their use in cartilage tissue engineering.

It is in this perspective that our previous research was based on the inhibition of the fibrotic process during neocartilage substitute generation, in vitro, with cells of human origin [29,40,45]. In these studies with human chondrocytes, in vivo experiments were realized in the nude mouse model by subcutaneous implantation of neocartilage substitutes. Despite the encouraging results obtained, this animal model is not the most relevant. Indeed, subcutaneous location does not present the same oxic conditions compared to articular cartilage. Furthermore, we observed, during these experiments, some invasion of mouse fibroblasts into the implant. To consider pre-clinical trials for the human model, we must perform the validation process in in vivo experiments in a larger mammalian animal. Cartilage diseases of the horse, the accessibility of its joints and its similar cartilage physiopathology compared to human, from a structural and cellular point of view, indicate that this animal represents a better mammalian model for in vivo implantation studies, compared to murine models. The main objective of the present study was to ensure the proof of concept in chondrocytes from horse origin, which also represents an excellent large animal model, concerning pathologies of the musculoskeletal system and articular disorders.

The use of an equine model comes with some technical limitations, such as the lack of molecular and biological tools compared to human research. Nevertheless, genome homologies between human and horse, and the high sequencing quality of *Equus caballus*, allows for the technological transfer between these two species [46]. Our present study highlights some subtle cellular differences during experiments. We have noticed that eAC numbers, and their viability after isolation from the cartilage, are substantially increased, as well as their proliferation, and the RNA and protein contents of the cells, when compared to human cells. During experiments, certain cell strains were amplified up to P16 without loss of proliferation. The high proliferation potential of eAC can represent an advantage in the MACI process that requires a great number of cells. Interestingly, cryopreservation of eAC at early passages is possible, while it remains more difficult for human AC, especially for those obtained from OA donors. These characteristics are not only explained by the use of different mammalian models, but more likely, by the homogeneity of the cartilage biopsies coming from healthy young or adult horses. This has the advantage of limiting cell variability related to cellular ageing, which is hardly possible in human research, since most of the samples are obtained after knee replacement by prosthetic surgery on aged subjects [47].

In this study, we have analyzed the impact of chondrocyte amplification on the cartilage ECM synthesis capacity of eAC. This cellular phenomenon is one of the most important limitations in the MACI procedure. We show that deep changes in phenotype are observed up to P3, particularly with the loss of chondrocyte marker expression. The monolayer amplification of eAC also induces type I collagen synthesis and catabolic events enhancement, as previously demonstrated in the princeps article [12,13]. Even if the *Col1a1* transcription is increased, type I collagen protein synthesis has not been demonstrated. One possible explanation is, that it has been seen that type I collagen expression appears after 21 days in confluent monolayer cultures like for hAC [13]. In our study, the time lapse between two passages is not of a sufficient magnitude to allow for type I collagen secretion, due to the rapid cell confluency reached by the eAC. It must be noted that *Col10a1* mRNA is only expressed during early passages. These observations can be explained by the fact that the cartilage biopsies were collected in order to maximize the number of cells harvested. Hypertrophic and superficial zones of articular cartilage were not split. Some traces of hypertrophic chondrocytes probably persist after cell isolation, but these cells enter in apoptosis during early amplification. Chondrocyte is a particular cell type that evolves in low oxygen tension. In addition to favoring the expression of chondrocyte markers, it has been shown that hypoxia inhibits terminal differentiation during chondrogenesis of MSC [23,24]. These findings can explain the absence of type X collagen expression during all our experiments.

During our chondrocyte redifferentiation experiments, we have used type I/III collagen scaffolds, a biomaterial already clinically used in MACI, but also in other tissue engineering therapies, including tooth, skin, and intestinal tissues [48,49,50]. Collagen sponges have the advantage of being easily adjustable to the thickness of the cartilage defect, and of preventing cellular loss by migration [51]. Moreover, it does not induce host reaction compared to type II collagen scaffolds [52], and 50% of this biomaterial is degraded after two weeks of implantation. Nevertheless, new advanced biomaterial innovations in MACI will have to take into account new functional supports which can interact with chondrocytes and control the delivery of growth factors or other biological actors [53].

To obtain the chondrocyte redifferentiation after monolayer cultures, 3D, and hypoxia, even though they both favor type II collagen synthesis [23], they do not allow for sufficient expression to lead to the conclusion that redifferentiation is optimal. We previously showed that IGF-I and TGF-β1 can be used to favor type II collagen expression in dedifferentiated chondrocytes, but they also induce hypertrophic and/or fibrotic markers of cartilage [45,54]. However, BMP-2 seems to remain the best candidate for significantly enhancing mature chondrocyte phenotype during redifferentiation. Type I collagen up-regulation induced by BMP-2, and the dedifferentiation process, can be limited by an RNA interference strategy, since transfection of a *Col1a1* siRNA can greatly enhance the matrix cartilage quality, by increasing the *Col2a1*/*Col1a1* as well as *Acan*/*Col1a1* ratios. The inhibition concerned preferentially the type I procollagen form that may chronologically affect mature forms at a longer term after implantation.

Difficulties have been encountered in *Col1a1* siRNA design and experiments. Due to the sequence homologies between *Col1a1* and *Col2a1*, the strong up-regulation of *Col1a1* forced us to perform a fine design of the siRNA. Thus, a total of five siRNAs have been designed and tested, and only one *Col1a1* siRNA proves to be specifically efficient. To better understand the observed problems, alignments between *Col1a1* siRNA sequences and mRNA secondary structure predictions have been realized (Figure S7). It seems that the ineffective siRNA targets steric hindrance zones, making RNA interference difficult. To maximize siRNA efficiency, the secondary structure of mRNA targets must be considered with great care [55,56,57].

Even though the *Col1a1* siRNA proves to be efficient in redifferentiated eACs, another relevant type I collagen mRNA interference could be developed for a significant increase in the mRNA knockdown. This interference should also be based on the *Col1a2* mRNA inhibition, concomitantly or not with the *Col1a1* knockdown, which takes into account the stoichiometric point of view. This hypothesis is based on the fact that proα1 chains of type I collagen cannot associate with proα2 chains if the 2:1 stoichiometry is not respected; homotrimers of proα1(I) chains will be degraded as a result of intracellular degradation. This process should lead to greater efficiency compared to *Col1a1* mRNA interference alone [58]. Other approaches may likewise be investigated, such as the use of imperfectly matched siRNA, which demonstrates a better mRNA target silencing [59].

Concerning the serine protease HtrA1, another undesirable marker which was over-expressed in osteoarthritis and associated with chondrocyte dedifferentiation, interestingly, we unambiguously and originally demonstrated that BMP-2 induces its down-regulation. No previous studies reported in the literature describe such an effect, and HtrA1 inhibition mediated by this chondrogenic factor was not observed in our previous study on hAC [29]. The differences between these two studies can perhaps be explained by the fact that the human chondrocytes used came from aged donors. Data from the literature demonstrated that TGF-β1 is able to induce HtrA1 [60], and that HtrA1 can interfere with BMP-2 signalling [61]^.^ Nevertheless, our current studies carried out on equine bone marrow MSCs show that the concomitant BMP-2/TGF-β1 treatment during chondrogenic differentiation induces a strong inhibition of HtrA1 protein synthesis compared with untreated MSCs [62], such as BMP-2 in the eAC of the present investigation.

*Htra1* siRNA interference, which was initially privileged because of the association of this serine protease to chondrocyte dedifferentiation and OA, was found to be highly effective. si*Htra1* inhibition, added to the BMP-2 effect on HtrA1 strongly limited its presence. In addition, due to the fact that HtrA1 can degrade hyaline cartilage ECM components, HtrA1 is indirectly implicated in pericellular matrix degradation of the chondrocyte [36], and induction of MMP-13 collagenase [60]. Even though the relationship between MMP-13 and HtrA1 cannot be significantly proved, analysis of each of our independent experiments seems to demonstrate that inhibition of HtrA1 by BMP-2 or *Htra1* siRNA always induces a parallel decrease of the steady-state amounts of *Mmp13* mRNA (Figure S8).

Even if the *Htra1* siRNA is efficient, and *Htra1* and *Col1a1* siRNA combination leads to a functional index increase, the use of this siRNA cocktail does not seem to be the most favorable strategy. Indeed, it appears that the use of both siRNA decreases all the ECM components of the neocartilage substitute, and provokes a partial loss of the BMP-2 benefit. An ambiguity in the BMP-2 effect on HtrA1 expression and *Htra1* inhibition on the global behavior of chondrocytes may be supposed, in the sense that Htra1 could play a bifunctional role in chondrocyte phenotype. Tiaden et al. have shown that HtrA1 plays a crucial role in osteogenesis by the positive regulation of matrix mineralization with its catabolic activity [33], and shows that the concentration of HtrA1 protein in chondrocytes near bone callus during new bone formation is important, suggesting its implication in both chondrogenesis and endochondral ossification. However, it remains to be established if the Htra1 RNA interference cannot specifically favor chondrocyte phenotype stabilization, as demonstrated in our present study, by preventing/counteracting its osteogenic positive effects in the context of bone repair.

Furthermore, investigation of experimental HtrA1 phenotype models, using in silico human database analysis by GenomeRNAi [63], shows that HtrA1 (PRSS11) can act as a down-regulator of the Wnt signalling pathway, following Wnt3A stimulation. This result has been extracted from RNAi screening studies on Wnt/β-Catenin in different cell lines [64]. This signalling pathway has been described as an important actor of MSC fate, BMP-2 signalling, and terminal differentiation of chondrocyte [65,66].

The data from the literature, along with our results on si*Htra1* use, suggest that this serine protease, other than/with the exception of ECM remodelling and TGF-β response modulation, may play a role in chondrocyte phenotype stabilization, or the differentiation status. It would be interesting to study the impact of HtrA1 inhibition on the induction of chondrogenesis in MSCs, to try to elucidate the functional role of this serine protease in the mature chondrocyte phenotype.

In conclusion, the use of an efficient *Col1a1* interference, alone or associated with *Htra1* interference, in the presence of BMP-2, clearly increases the cartilage quality by favoring type II collagen and aggrecan syntheses, while inhibiting type I collagen production in eAC, leading to a significant enhancement of the corresponding functional indexes, and reflecting the hyaline nature of the neocartilaginous ECM. Our study suggests that transient RNAi strategy can regulate the catabolic and anabolic components during the MACI procedure mediated by BMP-2 in the equine model. Our results will need to be further validated by establishing the proof of concept in preclinical trials in horse.

## 4. Materials and Methods

### 4.1. eAC Isolation and Cell Culture

eAC were isolated from cartilage biopsy of healthy equine carpal or femoral condyle joints from 10 young mature or adult horses (4–10 years). All procedures described in the present study were approved by the Ethics Committee for Animal Experimentation (ComEth ANSES/ENVA/UPEC, 94701 Maisons-Alfort, France; n° 15-023 (10 March 2015), n° 10-0051 (10 September 2014).

Healthy cartilage samples were cut into small slices (approximately 1–2 mm thickness, and 4–6 mm in diameter), and chondrocytes were isolated by sequential digestion for 45 min at 37 °C with 2 mg/mL of protease from *Streptomyces griseus* type XIV (Sigma-Aldrich, Saint Louis, MO, USA) for non-specific hydrolysis of ECM proteins, and then overnight at 37 °C with 2 mg/mL of type I collagenase from *Clostridium histolyticum* (Thermofisher, Waltham, MA, USA), which removes collagenic components of the ECM. Both were prepared with high glucose (4.5 g/L) Dulbecco’s modified Eagle’s medium (DMEM). The cell suspension was filtered through a 70 μM mesh nylon membrane, centrifuged, counted with trypan blue for viability evaluation, seeded in plastic vessels at a density of 2 × 10^4^ cells/cm^2^, and cultured in DMEM supplemented with 10% fetal bovine serum (FBS) (Thermofisher), 100 IU/mL of penicillin, 100 μg/mL of streptomycin, and 0.25 μg/mL of Fungizone, in an atmosphere of 5% CO_2_ at 37 °C. During expansion, the medium was changed twice a week. For cell passages, at 90% confluence, chondrocytes were harvested by trypsinization with 0.05% trypsin/1 mM EDTA (Thermofisher), counted with trypan blue for viability evaluation, and seeded again at 2 × 10^4^ cells/cm^2^ for the next passage.

### 4.2. eAC Dedifferentiation

eAC were amplified during 11 passages (P0–P11). For each passage, at 90% confluency, one cell culture flask was harvested for protein or RNA extraction, and the other for viability evaluation and cell counting. Equations used for doubling time determination was described by Martin Clynes [67]: time between two passages (h); *N*_0_: initial cells number; *N*_t_: cells number at the harvest; n: number of cells generation during; k: generation by time units; g: doubling time (h).

### 4.3. Cryopreservation

By contrast with human articular chondrocytes, the particularity of eAC lies in the possibility of cryopreservation. All chondrocytes cryopreservations were realized at P0 or P1. After counting, cells were centrifuged and suspended in cryopreserved medium (6 to 10 × 10^6^ cells/mL) composed of 90% FBS and 10% dimethyl sulfoxide (Sigma-Aldrich). Freezing was performed using CoolCell Cell Freezing Containers (Biocision, San Francisco, CA, USA) and stored in liquid nitrogen.

### 4.4. 3D Inclusion and Hypoxia Cell Culture

After thawing, cells were amplified up to P2. For redifferentiation studies, chondrocytes at P3 were included in scaffolds composed of native type I collagen (90–95%) and type III collagen (5–10%) from calf skin, manufactured by Symatèse Biomatériaux (Chaponost, France). Scaffolds were 2 mm thick and 5 mm in diameter. They were cross-linked using glutaraldehyde to increase their stability, and sterilized with β-radiation. Cell seeding into collagen sponges was performed by dropping 20 μL of the cell suspension on the scaffold (6 × 10^5^ cells/scaffold) in 96-well culture plates, dried for 1 h at 37 °C under 5% CO_2_, and incubated at 37 °C under 5% CO_2_ for 16 h with 3D medium composed of DMEM + 2% FBS + 50 μg/mL of l-ascorbic acid 2-phosphate sesquimagnesium salt hydrate (Sigma-Aldrich). The next day, scaffolds were transferred to 24-well plates, in 3D medium (600 μL/scaffolds) pre-equilibrated to 3% O_2_ by bubbling, with or without 50 ng/mL of BMP-2 (rhBMP-2, inductOs, Wyeth Europa Ltd., Gatwick, UK). This point established the day 0 (D0). Cells were incubated in hypoxia (2–4% O_2_) at 37 °C under 5% CO_2_ for 7 days. Hypoxic cultures were performed in Plas-Labs basic multi-station glove box (Sigma-Aldrich). The medium was changed twice a week.

### 4.5. siRNA Transfection and In Silico Analysis

Design of siRNA (Supplemental Table S1) was performed by Eurogentec service, and matching was confirmed using NCBI’s Basic Local Alignment Search Tool (BLAST). Sequences and BLAST results are presented in Figure 5A,B and represented with CLC Sequence Viewer 7.7 software (Qiagen, Manchester, UK). Mammalian siRNA duplexes’ negative control was used as non-targeting siRNA (Qiagen). eAC were seeded into type I/III collagen scaffolds, as described earlier. At D0, eAC were transfected with a mix (150 μL/scaffolds) of INTERFERin (Polyplus-transfection), OptiMEM (Thermofisher), and small interfering RNA (siRNA) for 10 min at room temperature, according to the manufacturer’s instructions. Quantities of 2, 5, 10, or 25 nM of siRNA were transfected. Then, the INTERFERin siRNA complex was slowly added to scaffolds disposed in 450 μL of 3D medium, pre-equilibrated to 3% O_2_ by bubbling, with or without 50 ng/mL of BMP-2. eAC were then cultured in hypoxia (2–4% O_2_) for 7 days. The first medium change for siRNA medium elimination was performed two days after transfection.

### 4.6. RNA Extraction and RT-qPCR Analysis

Total RNA was extracted using Trizol Reagent according to the manufacturer’s instructions. One microgram of RNA was reverse transcribed into cDNA using reverse transcriptase (MMLV, Thermofisher) and oligodT (Eurogentec, Seraing, Belgium). RT-qPCR was performed on StepOnePlus Real-Time system (Thermofisher) using Power SYBR Green PCR (Thermofisher). Sequences of the primers used are listed in Supplemental Table S1. β-actin gene (*Actb*) was used as an endogenous reference gene. Relative gene expression was analyzed using the StepOnePlus software and calculated using the 2^−∆∆*C*t^ method, or the standard curve method, depending on the efficiency of *Actb* amplification and each target gene, and expressed as the mean of triplicate samples [68].

### 4.7. Protein Extraction and Western Blots 

After treatment, scaffolds containing cells were rinsed once with ice-cold DPBS (Dulbecco’s Phosphate-buffered saline) (PAN-Biotech, Aidenbach, Germany), frozen, crushed and total proteins were extracted from cells using the RIPA (Radioimmunoprecipitation assay)-lysis buffer with protease inhibitors. Protein concentration was assessed according to the Bradford colorimetric assay (Bio-Rad, Hercules, CA, USA). A quantity of 15 μg of total proteins were separated into 10% polyacrylamide gels containing 0.1% SDS (Sodium dodecyl sulfate), and transferred to a polyvinylidene difluoride membrane (PVDF) (Millipore, Billerica, MA, USA). Unspecific binding sites of the membrane were blocked with 10% non-fat milk powder in Tris-buffered saline with 0.1% Tween (TBST) for 2 h. Membranes were incubated overnight at 4 °C with rabbit anti-human type I collagen (Novotec, Reuver, The Netherlands), rabbit anti-human type II collagen (Novotec), rabbit anti-human type X collagen (Abcam, Cambridge, UK), rabbit anti-human HTRA1 (Millipore), rabbit anti-human β-tubulin (Santa Cruz Biotechnology, Dallas, TX, USA), or rabbit anti-human GAPDH. The following day, membranes were washed three times, followed by an incubation with HRP-conjugated goat anti-rabbit IgG antibody (Jackson Immunoresearch, West Grove, PA, USA). Signals were visualized with the chemiluminescence method (ECL plus Western blotting detection reagent+, Santa Cruz Biotechnology) and developed on X-ray film (Santa Cruz Biotechnology).

### 4.8. Statistical Analysis

All experiments were replicated with cells from different specimens. Values are reported as means ± SD or box plots. To evaluate if biological samples answer to a normalcy law, Kolmogorov–Smirnov test was assessed using Graphpad Prism software (GraphPad Software, Inc., San Diego, CA, USA). Statistical analyses were performed using the Mann–Whitney *U*-test or unpaired *t*-test to determine any significant differences between two groups. Because the siRNA effect depends on the target basal expression of untreated control cases used for normalization, the Wilcoxon signed-rank test or paired *t*-test were also used. All statistical analyses were done using Prism v6 (Graphpad). The *p*-value of ≤0.05 was considered to be significant.

## Supplementary Materials

Supplementary materials can be found at www.mdpi.com/1422-0067/18/9/1842/s1.
