# Supplementary material for: RNA Interference and BMP-2 Stimulation Allows Equine Chondrocytes Redifferentiation in 3D-Hypoxia Cell Culture Model: Application for Matrix-Induced Autologous Chondrocyte Implantation
Source: Int J Mol Sci. 2017 Aug 24;18(9):1842. doi: 10.3390/ijms18091842 (PMC5618491; doi:10.3390/ijms18091842)
Supplement: Supplementary file 1 [file ijms-18-01842-s001.pdf]

**RNA interference and BMP-2 stimulation allows equine chondrocytes redifferentiation in 3D-hypoxia cell culture model: Application for matrix-induced autologous chondrocyte implantation.**

Rodolphe Rakic (1,2), Bastien Bourdon (1), Magalie Hervieu (1), Thomas Branly (1), Florence Legendre (1), Nathalie Saulnier (2), Fabrice Audigié (3), Stéphane Maddens (2), Magali Demoor (1), Philippe Galéra* (1).

1. Normandie Univ, UNICAEN, Laboratoire Microenvironnement Cellulaire et Pathologies (MILPAT), équipe Microenvironnement des Pathologies Dégénératives et Fibrotiques (MIPDF), EA 4652/BIOTARGEN EA 7450, Faculty of Medicine, Université de Caen Normandie, 14000 Caen, France.
2. Vetbiobank, Marcy l'Etoile, France.
3. Imaging and Research Centre of Equine Locomotor Disorders (CIRALE; Goustranville), Ecole Nationale Vétérinaire d'Alfort, Maisons-Alfort.

* Corresponding author: philippe.galera@unicaen.fr

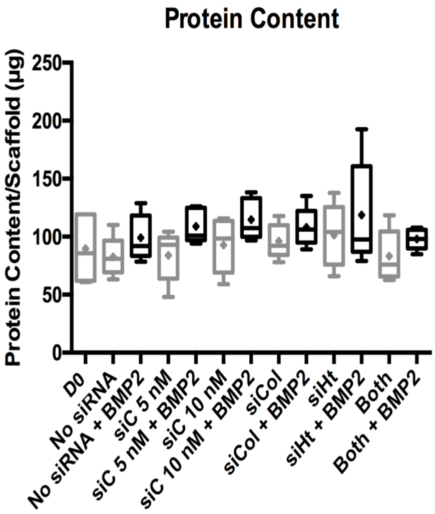


| | Conditions |
|---|---|
| D0 | Cells seeded in sponges and arrested after 16h of incubation |
| No siRNA | Cells not transfected |
| siC | Transfection with a negative control siRNA at 5 or 10 nM |
| siCol | Transfection with a *Col1a1* siRNA at 5 nM |
| siHt | Transfection with a *Htra1* siRNA at 5 nM |
| Both | Transfection with *Col1a1* and *Htra1* siRNA (5 nM + 5 nM) |

**Figure S1: Total protein content after treatments.** After eAC dedifferentiation during 2 passages, cells were trypsinized and seeded in type I/III collagen sponges. Cells were transfected or not by 5 nM of *Col1a1* siRNA (siCol), *Htra1* siRNA (siHt) or both (Both) in hypoxia and treated or not with BMP-2 (50 ng/ml) (+ BMP2) during 7 days. SiC represents the transfection of a negative control siRNA. Total protein extracts were determined by the Bradford protein assay. Box plots represent five independent experiments. Statistically significant differences were determined using the Mann Whitney test (*$p < 0.05$, **$p < 0.01$, ***$p < 0.001$).

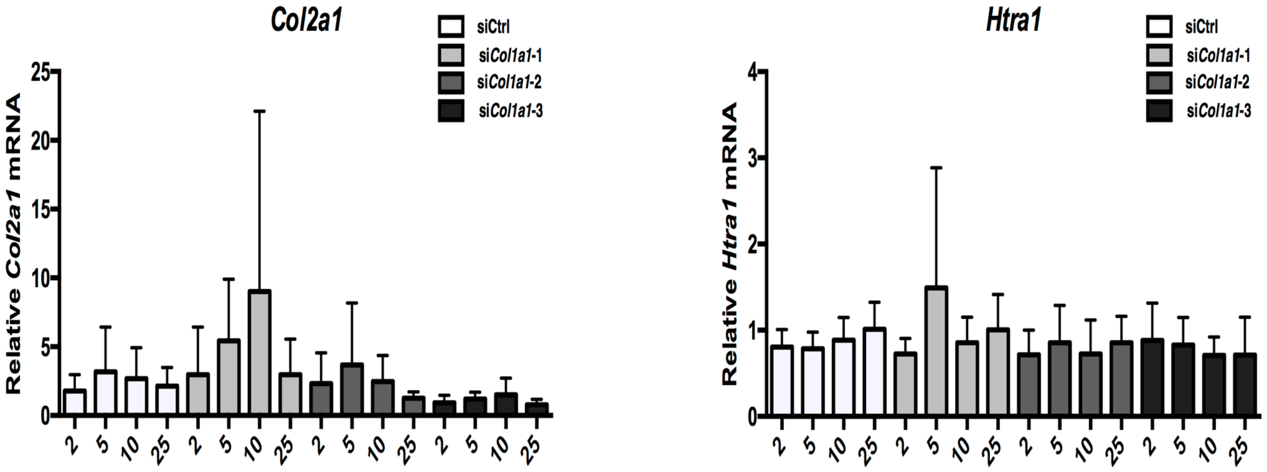


**Figure S2: Effect of *Col1a1* siRNA on *Col2a1* and *Htra1*.** Relative amounts of *Col2a1 and Htra1* mRNA were determined by RTqPCR. Cells transfected with a negative control siRNA were used as control (siCtrl). All the results are normalized versus BMP-2 treated cells without transfection, and presented as the relative expression of each gene. Box plots represent three independent experiments performed in triplicate. Statistically significant differences between siCtrl and transfected cells at the same siRNA concentration were determined using the Unpaired (*p < 0.05, **p < 0.01, ***p < 0.001) or the paired T-test ($^{\S}p < 0.05$, $^{\S\S}p < 0.01$, $^{\S\S\S}p < 0.001$).

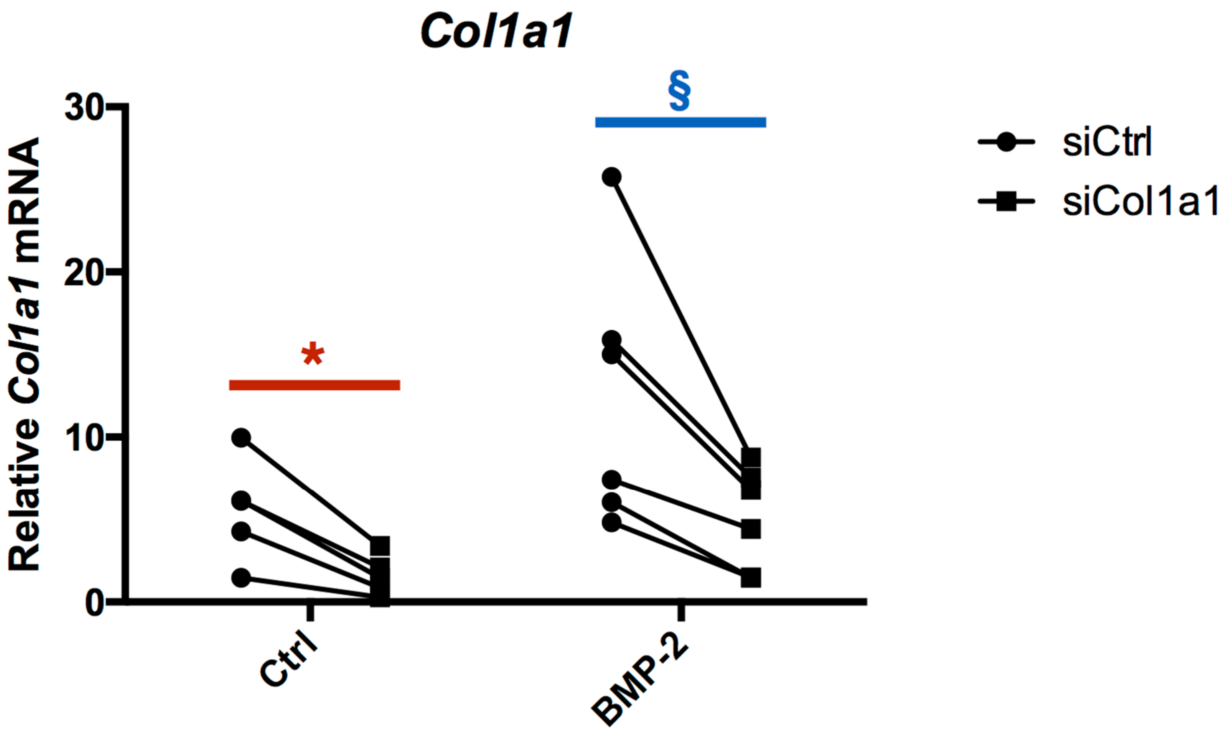


**Figure S3: Linear representation of si*Col1a1* effects on its target.** After eAC dedifferentiation during 2 passages, cells were trypsinized and seeded in type I/III collagen sponges. Cells were transfected with 5 nM of *Col1a1* siRNA (si*Col1a1*) in hypoxia and treated with BMP-2 (50 ng/ml) (BMP-2) or not (Ctrl) during 7 days. Relative mRNA levels of *Col1a1* were determined by RTqPCR. siCtrl represents cells transfected with a negative control siRNA. All the data are normalized versus eAC cultured in monolayer in normoxia, and presented as the relative expression of each gene. Box plots represent five independent experiments performed in triplicate. Statistically significant differences were determined using the Mann Whitney test (*p < 0.05, **p < 0.01, ***p < 0.001) or the Wilcoxon signed-rank test ($^{§}$p < 0.05, $^{§§}$p < 0.01, $^{§§§}$p < 0.001).

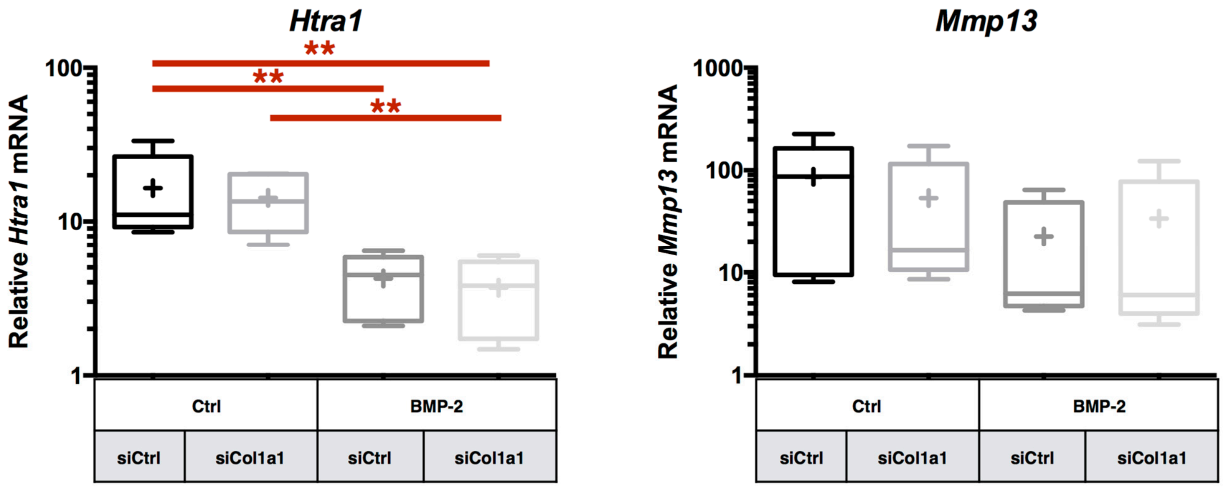


**Figure S4: *Htra1* and *Mmp13* mRNA expression during RNA interference targeting *Col1a1*.** After eAC dedifferentiation during 2 passages, cells were trypsinized and seeded in type I/III collagen sponges. Cells were transfected by 5 nM of *Col1a1* siRNA (si*Col1a1*) in hypoxia and treated with BMP-2 (50 ng/ml) (BMP-2) or not (Ctrl) during 7 days. Relative mRNA of *Htra1* and *Mmp13* were determined. siCtrl represents cells transfected with a negative control siRNA. All the results are normalized with eAC cultured in monolayer in normoxia, and presented as the relative expression of each gene. Box plots represent five independent experiments performed in triplicate. Statistically significant differences were determined using the Mann Whitney test (*$p < 0.05$, **$p < 0.01$, ***$p < 0.001$) or the Wilcoxon signed-rank test ($^{§}p < 0.05$, $^{§§}p < 0.01$, $^{§§§}p < 0.001$). Only the Mann Whitney test gives some significance.

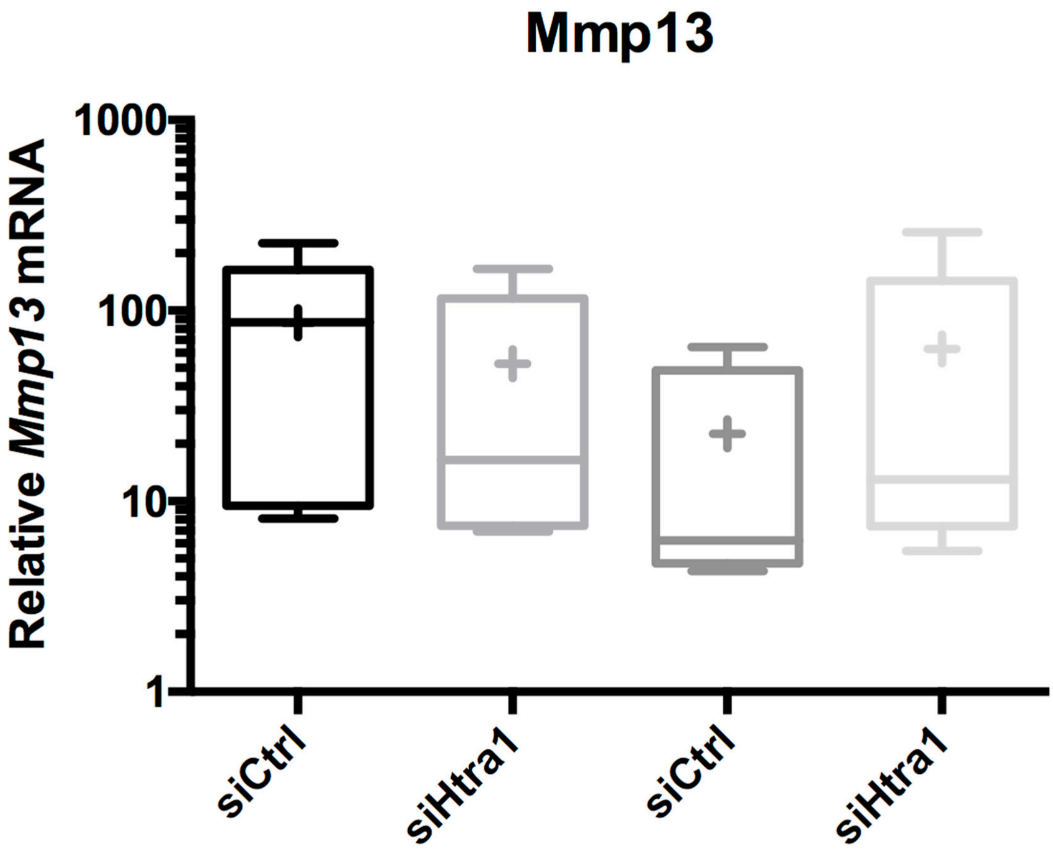


**Figure S5: *Mmp13* mRNA expression during RNA interference targeting *Htra1*.** After eAC dedifferentiation during 2 passages, cells were trypsinized and seeded in type I/III collagen sponges. Cells were transfected by 5 nM of *Col1a1* siRNA (si*Col1a1*) in hypoxia and treated with BMP-2 (50 ng/ml) (BMP-2) or not (Ctrl) during 7 days. Relative mRNA amounts of *Htra1* and *Mmp13* were determined. siCtrl represents cells transfected with a negative control siRNA. All the results were normalized versus eAC cultured in monolayer in normoxia, and presented as the relative expression of each gene. Box plots represent five independent experiments performed in triplicate. Statistically significant differences were determined using the Mann Whitney test (*$p < 0.05$, **$p < 0.01$, ***$p < 0.001$) or the Wilcoxon signed-rank test ($^{\S}p < 0.05$, $^{\S\S}p < 0.01$, $^{\S\S\S}p < 0.001$).

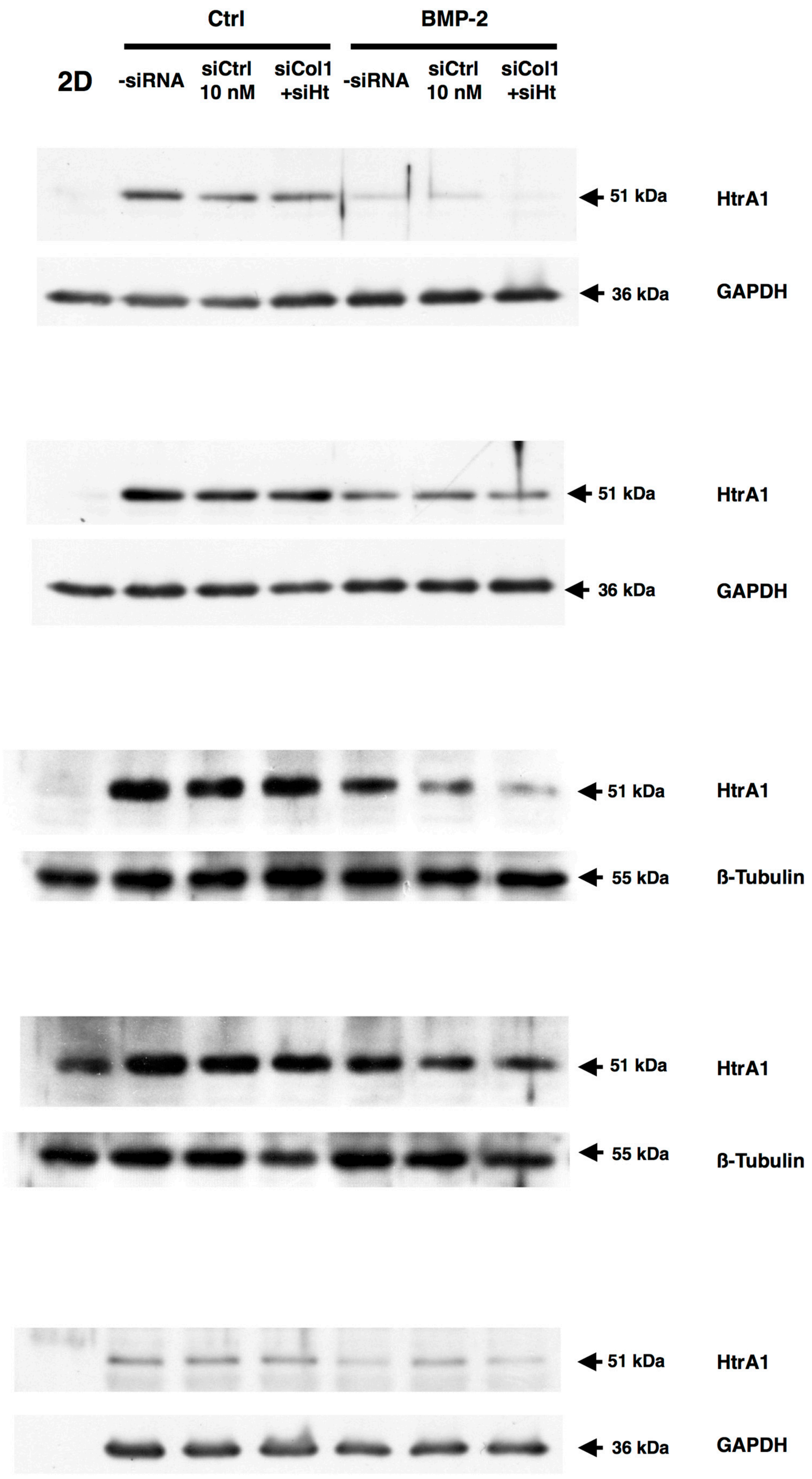
Ctrl
BMP-2
2D
-siRNA
siCtrl 10 nM
siCol1 +siHt
-siRNA
siCtrl 10 nM
siCol1 +siHt
51 kDa
HtrA1
36 kDa
GAPDH
51 kDa
HtrA1
36 kDa
GAPDH
51 kDa
HtrA1
55 kDa
ß-Tubulin
51 kDa
HtrA1
55 kDa
ß-Tubulin
51 kDa
HtrA1
36 kDa
GAPDH

**Figure S6: RNA interference targeting *Col1a1* and *Htra1* mRNA: Over-inhibition of Htra1 by the si*Htra1* treatment.** After eAC dedifferentiation during 2 passages, cells were trypsinized and seeded in type I/III collagen sponges. Cells were transfected by 5 nM of *Col1a1* siRNA (si*Col1*) or *Htra1* siRNA (*siHt*) in hypoxia and treated or not with BMP-2 (50 ng/ml) during 7 days. siCtrl represents cells transfected with a negative control siRNA. 2D : P3 eAC cultured in monolayer in normoxia. Protein extracts were analyzed in Western-blots for type II, type I, type X collagens, and HtrA1 versus GAPDH. Representative blots are shown (n = 5). Different levels of type II and type I collagen maturation forms are indicated such as type II procollagen (pro), with only C- or N- terminal propeptides (Pc/Pn) and the mature doubly cleaved form (mat). The 64 kDa type X collagen band represents signal peptide cleaved form.

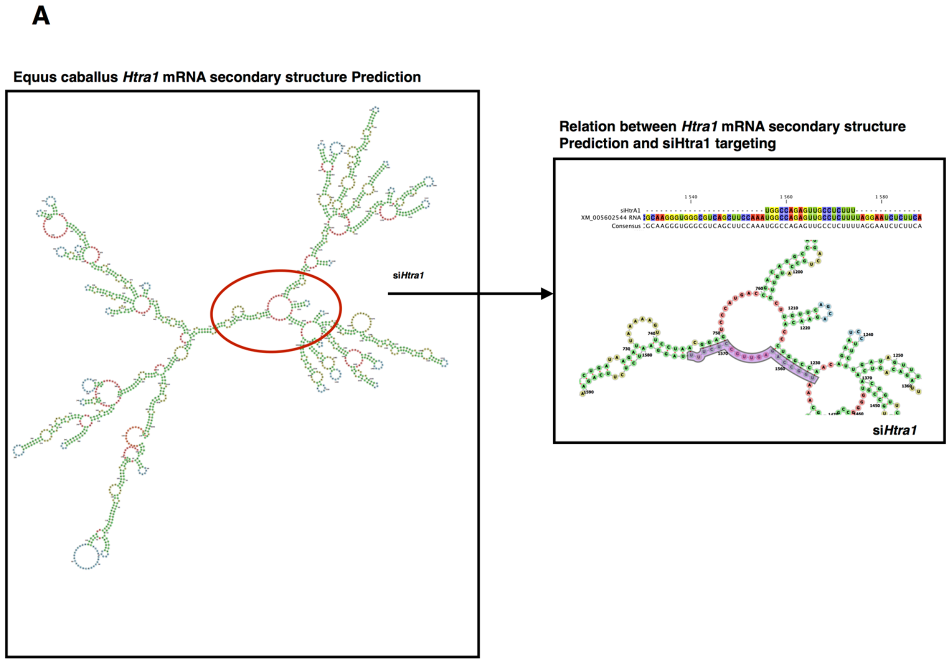
A
Equus caballus Htra1 mRNA secondary structure Prediction
siHtra1
Relation between Htra1 mRNA secondary structure Prediction and siHtra1 targeting
siHtra1

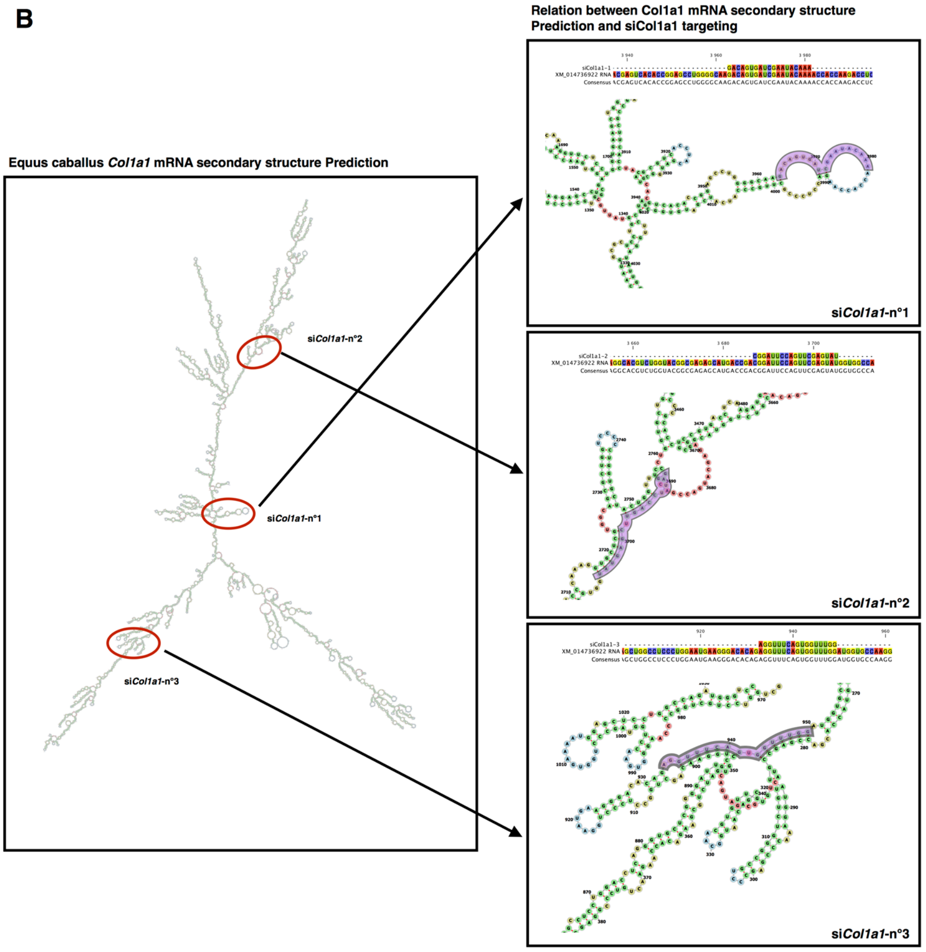
B
Equus caballus Col1a1 mRNA secondary structure Prediction
siCol1a1-n°2
siCol1a1-n°1
siCol1a1-n°3
Relation between Col1a1 mRNA secondary structure Prediction and siCol1a1 targeting
siCol1a1-n°1
siCol1a1-n°2
siCol1a1-n°3

**Figure S7: siRNA Effectiveness is correlated with RNA target secondary structure prediction.** mRNA secondary structure predictions were determined *in silico* using ViennaRNA Package 2.0 (Lorenz *et al.,* 2011[53]) with the minimum free energy prediction model. Sequence alignement between *Htra1* siRNA (**A**) or the three *Col1a1* siRNA (**B**) and their respective mRNA target. XM_005602544: Predicted Equus caballus HtrA serine peptidase 1 (HTRA1), mRNA NCBI reference sequence. XM_014736922: Predicted Equus caballus collagen, type I, alpha 1 (COL1A1), mRNA NCBI reference sequence. Software: CLC sequence viewer. The coloring of nucleotide is provided automatically by forna only software according to the type of structural element they are in (i.e. stem, interior, hairpin, multi or exterior loop).

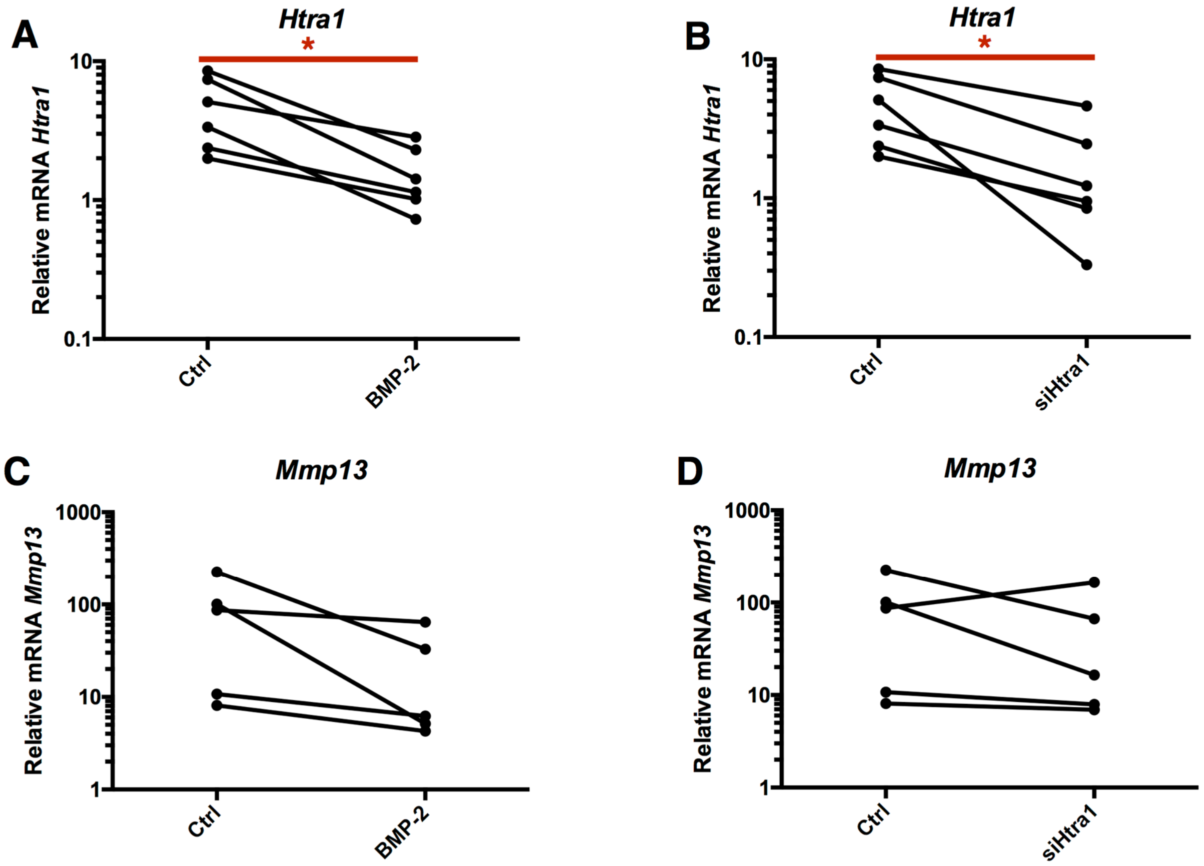


**Figure S8: Correlation between *Htra1* and *Mmp13* mRNA amounts under BMP-2 and *siHtra1* treatments.** After eAC dedifferentiation during 2 passages, cells were trypsinized and seeded in type I/III collagen sponges in hypoxia and treated with BMP-2 (50 ng/ml) (BMP-2) or not (Ctrl) during 7 days (**A**, **C**). During the culture, some cells were transfected or not with 5 nM of *Htra1* siRNA (*siHtra1*) (**B, D**). Relative mRNA of *Htra1* (**A, B**) and *Mmp13* (**C**, **D**) were determined by RTqPCR. Ctrl in panels **C** and **D** represent cells transfected with a negative control siRNA. All the results are normalized with eAC cultured in monolayer in normoxia, and presented as the relative expression of each gene. Box plots represent five independent experiments performed in triplicate. Statistically significant differences were determined using the Mann Whitney test (*$p < 0.05$, **$p < 0.01$, ***$p < 0.001$).

**A**

| siRNA duplex | Forward sequence 5'-3' |
|---|---|
| *Htra1* siRNA | UGGCCAGAGUUGCCUCUUU |
| *Col1a1* siRNA n°1 | GACAGUGAUCGAAUACAAA |
| *Col1a1* siRNA n°2 | CGGAUUCCAGUUCGAGUAU |
| *Col1a1* siRNA n°3 | GAGGUUUCAGUGGUUUGGA |

**B**

| Gene | Primer | sequence 5'-3' |
|---|---|---|
| *Actb* | forward | AGGCACCAGGGCGTGAT |
| | reverse | CTCTTGCTCTGGGCCTCGT |
| *Col1a1* | forward | TGCCGTGACCTCAAGATGTG |
| | reverse | CGTCTCCATGTTGCAGAAGA |
| *Col2a1* | forward | GGCAATAGCAGGTTCACGTACA |
| | reverse | CGATAACAGTCTTGCCCCACTT |
| *Col10a1* | forward | GCACCCCAGTAATGTACACCTATG |
| | reverse | GAGCCACACCTGGTCATTTTC |
| *Acan* | forward | ACACGGATGGTGTCCTCTTC |
| | reverse | CTCAGTCCACGGGTTACGAT |
| *Runx-2* | forward | GCAGTTCCCAAGCATTTCAT |
| | reverse | CACTCTGGCTTTGGGAAGAG |
| *Mmp13* | forward | TGAAGACCCGAACCCTAAACAT |
| | reverse | GAAGACTGGTGATGGCATCAAG |
| *Sox9* | forward | CAAGAAGGACCACCCGGACTA |
| | reverse | GGAGATGTGTGTCTGCTCCGT |
| *Alpl* | forward | GACATGACCTCCCAGGAAGA |
| | reverse | GCAGTGAAGGGCTTCTTGTC |
| *Htra1* | forward | GGACTTCATGTTTCCCTCAA |
| | reverse | GTTCTGCTGAACAAGCAACA |

**Table S1: Sequences list. A**: siRNA sequences used. **B**: Oligonucleotides used in RTqPCR experiments.
